# Single fraction ablative preoperative radiation treatment for early-stage breast cancer: the CRYSTAL study – a phase I/II clinical trial protocol

**DOI:** 10.1186/s12885-022-09305-w

**Published:** 2022-04-02

**Authors:** Maria Alessia Zerella, Mattia Zaffaroni, Giuseppe Ronci, Samantha Dicuonzo, Damaris Patricia Rojas, Anna Morra, Cristiana Fodor, Elena Rondi, Sabrina Vigorito, Francesca Botta, Marta Cremonesi, Cristina Garibaldi, Silvia Penco, Viviana Enrica Galimberti, Mattia Intra, Sara Gandini, Massimo Barberis, Giuseppe Renne, Federica Cattani, Paolo Veronesi, Roberto Orecchia, Barbara Alicja Jereczek-Fossa, Maria Cristina Leonardi

**Affiliations:** 1 Department of Radiation Oncology, European Institute of Oncology IRCCS, 20141 Milan, Italy; 2 Unit of Medical Physics, IEO, European Institute of Oncology IRCCS, Milan, Italy; 3 Radiation Research Unit, IRCSS, IEO European Institute of Oncology, Milan, Italy; 4 Division of Breast Radiology, IRCSS, IEO European Institute of Oncology, Milan, Italy; 5 Division of Breast Surgery, IEO, European Institute of Oncology IRCCS, Milan, Italy; 6 Department of Experimental Oncology, IEO European Institute of Oncology IRCCS, Milan, Italy; 7 Pathology Department, IEO European Institute of Oncology IRCCS, Milan, Italy; 8 Department of Oncology and Hemato-oncology, University of Milan, Milan, Italy; 9 Scientific Directorate, European Institute of Oncology IRCCS, Milan, Italy

**Keywords:** Single fraction preoperative radiotherapy, Early stage breast cancer, Stereotactic body radiation therapy, Clinical trial protocol

## Abstract

**Background:**

Breast-conserving surgery (BCS) and whole breast radiation therapy (WBRT) are the standard of care for early-stage breast cancer (BC). Based on the observation that most local recurrences occurred near the tumor bed, accelerated partial breast irradiation (APBI), consisting of a higher dose per fraction to the tumor bed over a reduced treatment time, has been gaining ground as an attractive alternative in selected patients with low-risk BC. Although more widely delivered in postoperative setting, preoperative APBI has also been investigated in a limited, though increasing, and number of studies. The aim of this study is to test the feasibility, safety and efficacy of preoperative radiotherapy (RT) in a single fraction for selected BC patients.

**Methods:**

This is a phase I/II, single-arm and open-label single-center clinical trial using CyberKnife. The clinical investigation is supported by a preplanning section which addresses technical and dosimetric issues. The primary endpoint for the phase I study, covering the 1st and 2nd year of the research project, is the identification of the maximum tolerated dose (MTD) which meets a specific target toxicity level (no grade 3–4 toxicity). The primary endpoint for the phase II study (3rd to 5th year) is the evaluation of treatment efficacy measured in terms of pathological complete response rate.

**Discussion:**

The study will investigate the response of BC to the preoperative APBI from different perspectives. While preoperative APBI represents a form of anticipated boost, followed by WBRT, different are the implications for the scientific community. The study may help to identify good responders for whom surgery could be omitted. It is especially appealing for patients unfit for surgery due to advanced age or severe co-morbidities, in addition to or instead of systemic therapies, to ensure long-term local control. Moreover, patients with oligometastatic disease synchronous with primary BC may benefit from APBI on the intact tumor in terms of tumor progression free survival. The study of response to RT can provide useful information about BC radiobiology, immunologic reactions, genomic expression, and radiomics features, to be tested on a larger scale.

**Trial registration:**

The study was prospectively registered at clinicaltrials.gov (NCT04679454).

## Background

Whole breast radiation therapy (WBRT) after breast-conserving surgery (BCS) is the standard of care for patients with early-stage breast cancer (BC) [1]. Over the last two decades, radiotherapy (RT) for BC has undergone major changes in fractionation, techniques and target volumes [2–4].

Hypofractionation is now considered the preferred schedule for adjuvant WBRT on the basis of the results of dedicated randomized phase III studies showing equivalent tumor control, improved acute toxicity and similar late toxicity rates compared to conventional fractionation [5–7]. Hypofractionation has been successfully applied in the setting of accelerated partial breast irradiation (APBI) where the treatment is given only to the tumor bed as the region at higher risk of relapse [8–10]. A number of APBI phase III trials using different techniques have showed that, in selected patients, APBI achieved a satisfactory local control, comparable with that of WBRT, but with fewer side effects, greater convenience, better quality of life, reduced costs [11–15]. The safety and the efficacy of APBI delivered in postoperative setting have stimulated investigations in the preoperative field as well. In this context, preoperative APBI can be regarded as anticipated boost, followed by WBRT after surgery, or can be used with neoadjuvant purpose, to reduce the size of the lesion allowing more conservative surgery and to test the tumor sensibility. The latter investigation might open to the possibility of a definitive RT with curative intent. Older and/or frail women that are less likely to receive standard of care treatments, often because of medical comorbidities, might benefit the most [16–18].

The algorithm to replace surgery with RT has proved to be successful in the treatment of oligometastases. Short courses of high dose stereotactic ablative radiosurgery (SRS) have frequently being used as alternatives to surgery for oligometastases. Starting from the benefits observed for solitary brain metastases, the use of SRS has been expanded to include extracranial sites. Scorsetti and coll. [19], considering a cohort of 33 BC oligometastatic patients, observed local control rates of 98 and 90% at 1 and 3 years, with complete and partial response rates of 53 and 34% respectively. These results may provide the rationale to investigate the effect of delivering high-dose single fraction to small primary BC.

The feasibility and the efficacy of a pre-operative radioablation approach on primary BC have been investigated in few clinical studies, using different technique, dose/fraction, number of fractions, total dose and irradiated volumes. Preliminary reports demonstrate low toxicity and a rate of pathological response worthwhile exploring further [20–27].

The aim of the present study is to test the feasibility, safety and efficacy of preoperative RT in a single fraction for selected BC patients.

## Methods/design

### Aim, design, and setting of the study

This is a monocentric phase I/II, single-arm and open-label trial planning to enroll a maximum of 79 patients over 5 years and using CyberKnife to deliver the ablative dose (18–24 Gy) to the tumor before surgery. The clinical investigation includes a preplanning section in which technical issues (contouring, set-up, dosimetry, treatment delivery, etc.) are addressed. The project was registered at clinicaltrials.gov (NCT04679454) and was approved by institutional Ethical Committee (identification number 1308).

### Study population

#### Participant characteristics and eligibility criteria

Inclusion and exclusion criteria are summarized in Table 1.

**Table 1:** Inclusion and exclusion criteria

ELIGIBILITY CRITERIA	
**INCLUSION CRITERIA**	Histologically proven unifocal adenocarcinoma of breast cancer
	cT1-cT2 cN0
	Age ≥ 18 years old
	Good general condition (ECOG 0–2)
	Planned surgery (BCS or mastectomy)
	Written informed consent
**EXCLUSION CRITERIA**	Tumor too close to skin or chest wall
	Pure non-invasive tumor
	Prior RT to the chest
	Neoadjuvant chemotherapy
	Coagulation, connective, autoimmunitary disorders
	Previous malignancies

*List of abbreviations*: *BCS* breast conserving surgery, *ECOG* Eastern Cooperative Oncology Group, *RT* radiotherapy

After study enrollment, a core biopsy is performed for tumor characterization. At the time of core biopsy, at least 3 gold fiducial markers, of 1 mm × 1.5 mm size and compatible with any radiologic examinations, are placed around the tumor under ultrasound guidance for RT target localization along the 3 axes.

Radiological work-up is completed with preoperative magnetic resonance imaging (MRI) in supine and prone position.

### Study design

The research project comprises 6 tasks:

1. Technological assessment – a pre-clinical investigation of radioablation feasibility using CyberKnife is conducted to establish protocol procedures;
2. Patients enrollment and monitoring - patients are enrolled according to the inclusion criteria. Maximum acute toxicity at 1 and 2 weeks after radioablation is evaluated to modulate the accrual throughout the phase I dose escalation study;
3. Histopathologic analysis - tumor characteristics and radiation response are investigated through histopathologic analyses of biopsy and surgical specimen;
4. Radiology and Radiomics tasks - imaging features extracted from pre- and post- radioablation MRI are quantitatively and semi-quantitatively analyzed to identify imaging markers of radiation response;
5. Molecular pathology – expression of genes involved in tumor–immune system interactions is analyzed through next generation sequencing (NGS) assays.

#### Phase I study

Dose escalation is designed as a traditional 3 + 3 rule-based study. This study design is the prevailing method for conducting phase I cancer clinical trials since it avoids the selection of phase II doses that cause a treatment-limiting toxicity in more than 17% of subjects [28]. This rule-based design proceeds with cohorts of three patients testing three dose levels: 18 Gy, 21 Gy and 24 Gy. Starting from the lowest prescription dose, if none of the three patients in the first cohort experiences a dose limiting toxicity (DLT), other three patients will be treated at the next dose level. However, if one of the first three patients experiences a DLT, three more patients will be treated at the same dose level. The dose escalation continues up to the 24 Gy dose level until at least 2 patients among a cohort of 3 to 6 patients experience DLT. The recommended dose for subsequent phase II trial is defined as the dose level just below this toxic value. Maximum tolerated dose (MTD) is decided when 6 patients are treated at a dose level and a maximum of 1 out 6 (17%) patients experiences DLT. The starting dose level of 18 Gy is considered to be safe based on the basis of previous studies [22]. Phase I study will enroll a maximum of 18 patients, depending on the number of dose level escalations and the number of DLTs observed.

#### Phase II study

The recommended dose level found in the first phase will be delivered in the phase II study. The primary endpoint of the phase II study is the rate of pathological complete response (pCR). In the current research project, radioablation is expected to be as effective as a single drug agent. A pCR rate of 20% is chosen to test the efficacy of radioablation.

A study requires 61 patients to decide whether the proportion of responders, i.e. P, is ≤9% or ≥ 20%. If the number of responses is 10 or more, the hypothesis that *P* ≤ 9% is rejected with a target error rate of 5%. If the number of responses is 9 or less, the hypothesis that *P* ≥ 20% is rejected. Therefore, in the projected population of 61 patients, at least 10 patients with pCR are required to reject the null hypothesis of no treatment efficacy.

### Treatment planning

The preoperative MRI is acquired in prone position for diagnostic purpose to exclude multicentricity. With the aim of improving target definition and tumor tracking during treatment, a preoperative MRI with fiducial markers in place in supine position is used for rigid and/or deformable registration with treatment planning non-contrast computed tomography (CT) images.

Gross tumor volume (GTV) is contoured by a radiation oncologist and then double-checked by a second radiation oncologist. The GTV is expanded by 0.5 cm to create the clinical tumor volume (CTV) which coincides with planning tumor volume (PTV). Plans are elaborated on the Cyberknife Treatment Planning System (Precision, Accuray), using a non-coplanar and non-isocentric approach. Treatment delivery was carried out using a real-time fiducial-based target tracking (Synchrony®): the total system accuracy of the Robotic stereotactic radioablation/motion tracking has been reported as < 1 mm [29–31], which allowed the use of much smaller margins compared with conventional radiosurgery and therapy methods.

Pre-specified dose constraints for organs at risk and planning objectives for target are based on the National Surgical Adjuvant Breast and Bowel Project B-39/Radiation Therapy Oncology Group 0413 partial breast trial [32] and on stereotactic radiosurgery [33].

Further optimization of dosimetric parameters will be done as the study proceeds.

### Treatment

Radioablation is performed within 4 weeks since patient entering the study and no systemic therapy is allowed in the pre-operative settings.

Surgical tumor removal is scheduled within 8 weeks from radioablation.

All patients receive postoperative RT without boost to the tumor bed, preferably with hypofractionation (preferably, 2.67 Gy × 15 fractions). Systemic therapy is administrated according to the institutional guidelines.

Post-surgical complications as well as radiation side effects are collected and follow-up is scheduled on regular basis throughout 3 years.

**Fig. 1:**
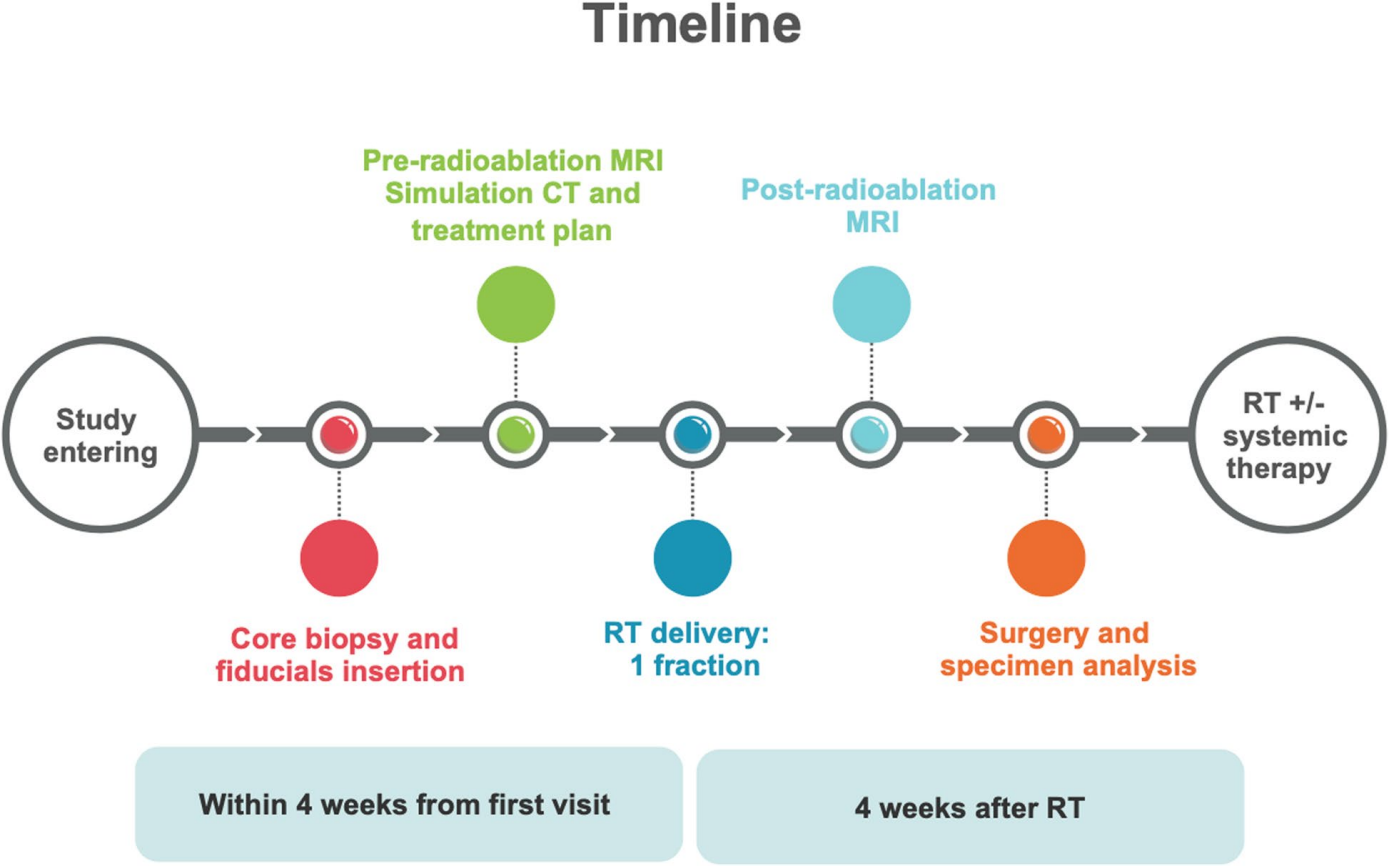
Overview of the study workflow

An overview of the treatment workflow is reported in Fig. 1.

### Endpoints of the study

#### Phase I endpoints

Primary endpoint of the phase I study is to identify MTD, which is the dose that meets a specific target toxicity level (no grade (G) 3–4 toxicity).

Outcome measures is obtained through acute skin/soft tissue toxicity, measured according to NCI CTCAE v. 4.03 [34]. Any G3–4 toxicity related to radioablation is considered dose limiting (DLT).

Secondary endpoints include chronic toxicity, cosmesis, post-surgery complications, outcomes of survival and relapse, extra-cutaneous complications and pCR.

#### Phase II endpoints

Primary endpoint is the evaluation of the efficacy measured in terms of pCR rate.

Outcome measures is obtained through the pCR rate after surgery, according to the Modified Residual Cancer Burden (RCB) index.

Secondary endpoints include acute/chronic toxicity, cosmesis, post-surgery complications, outcomes of survival and relapse, extra-cutaneous complications.

### Ethical aspects

The study is conducted according to the Declaration of Helsinki/Tokyo and to Good Clinical Practice guidelines.

The protocol has been presented to and approved by the ethics committee of the European Institute of Oncology IRCCS, Milan. After complete explanation of the objectives and modalities of the study, each patient is required to give written informed consent for participation in the study.

## Discussion

A broader knowledge and better understanding of BC biology along with advances in RT planning, targeting, and delivery, have changed the attitude towards BC treatments, with the abandonment of the concept of “one size fits all” in favor of approaches more tailored on the tumor and on the individual. Escalation and de-escalation of RT dose and target volumes, as well as RT omission, must be weighed up at the cost-benefit analysis. This approach expresses the modern concept of high-precision medicine where decision-making is processed at a multidisciplinary level, in accordance to the biologic aggressiveness of the disease, the radiosensibility, the expected pattern of relapse and the integration with systemic therapies.

More recently, the hypothesis to anticipate RT before surgery has become a growing area of investigation. Preoperative RT is not a new concept in itself, as early experiences date back to the ‘70s. However, the historical approach typically encompassed the whole breast and the regional nodal drainage and the extension of the RT fields drew some concerns regarding the increase of surgical complications [35]. Therefore, preoperative RT fell out of use as the neoadjuvant systemic therapy was gaining ground. With the introduction and affirmation of APBI, which in the postoperative setting has been proven to be an acceptable alternative to WBRT in properly selected patients [11–15, 32, 33], the interest for preoperative RT has grown again. Preoperative APBI carries a number of advantages compared to postoperative APBI [36]. By irradiating an intact lesion with an image-guided approach, issues related to treatment volume delineation are minimized, decreasing inter-observer contouring variability and improving target volume definition thanks to the co-registration of MRI with simulation CT images. As the target corresponds to the tumor rather than to a large postoperative area, the irradiation of uninvolved breast tissues is significantly reduced, allowing ultra-hypofractionated schedules and further shortening RT duration. In addition, as consequence of cytotoxic effect, the tumor may shrink, reducing the amount of normal tissue to be removed during the surgery. The combination of more conservative surgery and smaller irradiated volume might result in milder breast fibrosis and in an overall better cosmesis. Moreover, preoperative APBI provides the possibility to test the radio-sensitivity analyzing tumor changes at molecular and genomic levels. These data can ideally be employed to build a classifier to stratify class of responders and non-responders, contributing to a better understanding of radiobiologic effects and behavior of the irradiated tumors [23] in a wider view of personalized medicine. Also MRI quantitative parameters collected in pre- and post- radiation setting could potentially be applied as radiation response biomarkers [37] and prognostic/predictive tools [38]. Researchers at the Duke Cancer Institute [22] extensively investigated the effect of preoperative single fraction RT in the field of imaging and gene expression and found an increase in vascular permeability and a decrease of cellular density after radioablation in MRI images. The current study provides the opportunity to compare pre- and post- radioablation MRI images in order to assess radiological tumor response and identify imaging biomarkers of radiation response through qualitative and semiquantitative analysis. The ultimate goal is to identify those tumors which achieve complete response and that may remain in control without the need of being surgically removed.

As observed for postoperative APBI, different techniques have been used to perform preoperative APBI: three-dimensional conformal RT (3D-CRT), intensity modulated RT (IMRT), volumetric-modulated arc-therapy (VMAT), SRS and stereotactic body RT (SBRT), brachytherapy (BRT) and proton beam therapy (PBT) [39–43]. Main preoperative APBI studies published in the literature are summarized in Table 2, while Table 3 and Table 4 describe the ongoing clinical trials. Because of the complexity of BRT and the requirement of a larger PTV for 3DCRT/IMRT, stereotactic techniques are preferred for preoperative approach to BC, given their proven efficacy and safety in the treatment of a great variety of primary and metastatic tumors [44–47]. In the current study, single fraction preoperative radioablation is investigated using robotic SBRT with CyberKnife system, which allows smaller margins, less radiation exposure to adjacent normal tissue and, potentially, better cosmetic outcomes. Moreover, Cyberknife is capable of tracking the target volume during irradiation to achieve high conformity and dose gradient to the tumor. The current research project plan focuses first on the identification of the maximum tolerated dose, whose efficacy is investigated in terms of pCR rate in the phase II study. The starting dose is 18 Gy in single fraction, which can be considered relatively safe. This dose level was also used by Horton et al. [22] in their phase I dose escalation trial of single-dose preoperative RT for early stage unifocal BC patients. Dose escalation levels were 15 Gy (*n* = 8), 18 Gy (n = 8) or 21 Gy (*n* = 16), delivered with IMRT. Post-operative conventional RT was administered to patients not satisfying the eligibility criteria following surgical resection (*n* = 3). No dose-limiting toxicity was observed up to 21 Gy and, at a median follow-up of 23 months, no evidence of tumor progression was documented. Physician-rated cosmetic outcomes were good/excellent and chronic toxicities were low in patients receiving preoperative RT only. Among the 3 patients receiving post-operative RT, one with a connective tissue disorder developed two grade 3 chronic toxicities, and one with diabetes suffered from wound infection, while all of them presented a fair/poor cosmetic outcome. Another phase I dose-finding SBRT trial was conducted by Bondiau et al. [21], where 5 dose levels (from 19.5 Gy to 31.5 Gy in 3 fractions) were tested, using a robotic SBRT system during the course of preoperative chemotherapy in locally advanced BC patients. Surgery and conventional post-operative RT were performed 6–8 weeks after neoadjuvant chemoradiotherapy. After observing only one grade 3 toxicity (dermatologic in nature) at 28.5 Gy, the authors concluded that the lower dose of 25.5 Gy in 3 fractions could be used for the phase 2 trial. The French series showed high rate of pCR (36%) and breast-conserving surgery (92%). These excellent results were likely due to the synergic and additive effect of concomitant radiochemotherapy. Relying on RT alone, the rate of pCR is expected to be lower, ranging from 10 to 15%. In the abovementioned series, all patients received WBRT without boost to the tumor bed and none of them presented any excess of toxicity, despite the fact that preoperative APBI was delivered concomitantly with neoadjuvant chemotherapy. This favorable outcome is in contrast to the fair/poor cosmesis of the patients enrolled by Horton and coll., who received both pre- and post-operative RT [22]. Horton and coll. Explained such an inconsistency between the 2 series with the presence of comorbidities, affecting the tolerability to the treatment, and with differences in fractionation schedules.

**Table 2:** Summary of the studies on preoperative breast cancer radiotherapy

Author and year	Inclusion criteria	n of pts	Treatment	Systemic therapy	Time to surgery	Postoperative RT	Outcomes	Toxicity	Follow-up (months)
Bondiau et al., 2013 [21]	Unifocal BC not suitable for BCS, HER2 negative	26	Robotic SBRT (Cyberknife)/19,5–31.5 Gy/3 fr (5 dose levels)	CHT	4–8 weeks after last CHT	Yes, 3D-CRT	pCR 36% (NS) 92% BCS rate 96% ORR	none	30
Horton et al., 2015 [22]	Age > 55 years, T1 BC or low- intermediate DCIS ≤2 cm, cN0, ER + and/or PR+, HER2-	32	IMRT/15–21 Gy (3 dose levels)/1 fr	none	within 10 days after RT	Yes (Only to patients not satisfying eligibility criteria after BCS)	Significant increase in MRI post- radiation vascular permeability and decreased cellular density	13 Grade2; 2 Grade 3	23
van der Leij et al., 2015 [23]	Age > 60 years, invasive, unifocal BC ≤ 3 cm on MRI, non-lobular, negative SNB	70	3D-CRT or IMRT or VMAT/40 Gy/10 fr	none	6 weeks after RT	No	2 local recurrences	11% mild-moderate 23 induration at 12 months; 2% mild-moderate fibrosis at 24 months	23
Nichols et al., 2017 [24]	Unifocal invasive BC < 3 cm at mammography or MRI, cN0	27	3D-CRT /38.5 Gy/10 fr (twice daily)	none	> 21 days after RT	No	pCR 15% (NS) ORR 88.9%; Ki-67 reduction after RT in 70.4%	PRCO fair and poor in 17 and 5% at 1 year, respectively	43
Tiberi et al., 2020 [27]	postmenopausal status, age > 65 years, stage I (cT1N0) invasive BC, unifocal luminal A, ER+, her2-negative, G1–2	10	SBRT 20 Gy/1fr	none	11–13 weeks after RT	Yes if: Grade 3, lymphovascular invasion, pT4, triple neg, extensive DCIS, tumor size > 3 cm, lobular histology	pCR: 0 (median residual cellularity was 3% in 8 patients; for the other 2 patients no response at all was observed)	none	< 12
DOSIMETRIC STUDIES									
Charaghvandi et al., 2015	Tumor size up to 30 mm, scheduled for BCS and WBI	20	IMB and VMAT 15 Gy/1 fr + integrated ablative boost of 20 Gy	none	–	–	Dosimetrically feasible with IMB and VMAT	–	–
Yoo et al., 2015 [44]	Patients enrolled on a preoperative, dose escalation, single fraction PBI clinical protocol	16 (8 + 8)	3D-CRT, non-coplanar IMRT, coplanar IMRT, VMAT 15 Gy/1fr and 18 Gy/1 fr	none	–	–	IMRT plans provided homogeneous and conformal target coverage, skin sparing, and short delivery time	–	–

*List of abbreviations: 3D-CRT* 3D conformal RT, *BC* breast cancer, *BCS* breast conserving surgery, *CHT* chemotherapy, *CTV* clinical target volume, *DCIS* ductal carcinoma in situ, *FX* fraction, *IMB* interstitial multicatheter brachytherapy, *IMRT* intensity modulated RT, *MRI* magnetic resonance imaging, *PBI* partial breast irradiation, *pCR* pathological complete response, *RT* radiotherapy, *SBRT* stereotactic body radiotherapy, *VMAT* volumetric modulated arc therapy, *ER* estrogen, *PR* progesterone, *HER2* human epidermal growth factor receptor 2, *SNB* sentinel node biopsy, *NS* not statistically significant, *ORR* objective response rate, *PRCO* patient reported cosmetic outcome

**Table 3:** Summary of the clinical trials regarding pre-operative breast cancer radiotherapy in more than one fraction

Trial ID	Status	Title	Treatment	Description/endpoint	Primary outcome measure	Estimated/ actual primary completion date
NCT04360330	Recruiting	SABER study for selected early-stage BC	Stereotactic Ablative RT RT dose: 4 predefined dose levels (35 Gy, 40 Gy, 45 Gy, 50 Gy, in 5 fractions given on non-consecutive days, over a period of 2 weeks). Then standard of care surgery.	Find the most effective RT dose to give to BC in a shorter period of time, prior to surgery. Toxicity, cosmesis and quality of life will be assessed.	Find the recommended Phase 2 Dose of Pre-Operative SABER in terms of the highest dose level tested for which no more than 1 out of 6 patients experience DLT	August 2023
NCT04234386	Recruiting	GammaPod Dose Escalation RT for early-stage BC	GammaPod Radiation RT dose: 4 predefined dose levels (21 Gy, 24 Gy, 27 Gy, 30 Gy). Then standard of care surgery.	Determine a safe and effective dose of pre-operative RT to treat early stage BC. Cosmesis and quality of life will be assessed.	Establish the single-fraction MTD and DLTs	December 2025
NCT03624478	Recruiting	Hypofractionated RT in Treating Participants with BC before surgery	Hypofractionated RT RT dose: NS (5 consecutive days followed by standard of care surgery).	Assess the efficacy, toxicity, cosmetic outcome and pathologic changes of hypofractionated RT in treating breast cancer before surgery (phase II trial)	pCR	August 17, 2021
NCT03043794	Recruiting	Study of Stereotactic RT for BC	Stereotactic RT RT dose: 21 Gy followed by standard of care surgery.	Assess the efficacy, toxicity, cosmetic outcome, quality of life, and translational correlates to preoperative stereotactic RT for low risk BC (phase II trial)	pCR	August 1, 2022

*List of abbreviations: BC* breast cancer, *RT* radiotherapy, *MTD* maximum tolerated dose, *DLT* dose limiting toxicity, *pCR* pathological complete response, *NS* not stated

**Table 4:** Summary of the clinical trials regarding pre-operative breast cancer radiotherapy in single fraction

Trial ID	Status	Title	Treatment	Description/endpoint	Primary outcome measure	Estimated/ actual primary completion date
NCT03863301	Recruiting	MRI-guided single dose preoperative RT in low-risk BC	MR-guided single dose preoperative PBI Dose: a single dose of 20 Gy to GTV and 15 Gy to CTV (GTV + 20 mm margin). Breast conserving surgery will be performed 12 months following PBI.	Evaluate efficacy of the treatment 12 months after RT, and to collect data on response monitoring (MRI, liquid biopsies and biopsy of the irradiated tumor). Patient-reported outcome measures will be evaluated.	pCR	November 2022
NCT02482376	Recruiting	Preoperative single-fraction RT in early-stage BC	Stereotactic body RT Dose: a single fraction of 21Gy. Then standard of care surgery.	Assess toxicity, efficacy and to provide an avenue for understanding breast cancer radiation response through pre- and post-radiation breast tumor samples	Physician reported rates of good/excellent cosmesis at baseline and 6 months, 1, 2, and 3 years post-treatment as measured by the NRG cosmesis scale	September 2021
NCT03520894	Recruiting	RT in preoperative setting with CyberKnife for BC	Single fraction of RT with Cyberknife Dose: a single fraction of 21 Gy.	Evaluate the safety and feasibility of single fraction RT and to identify predictive factors for outcome based on biologic and clinical parameters	Rate of acute skin toxicity events, measured according to RTOG/EORTC scale	May 1, 2022
NCT01717261	Recruiting	Single Pre-Operative RT (SPORT) for low-risk BC	Single fraction Pre-Operative RT Dose: Dose escalation (15 Gy, 18 Gy, 20 Gy).	Assess if RT administered in a single preoperative fraction is tolerable in terms of acute, chronic toxicity and cosmetic outcome. Ipsilateral BC recurrence at 5 years will be assessed.	Acute toxicity and wound healing complications from the preoperative radiation treatment as per NCI CTCAE Common Toxicity Scale.	December 2019
NCT02316561	Completed	Single Dose Ablative RT for Early-Stage BC	Single dose ablative PBI Dose: NS. Surgery will be performed 6 months after the ablative RT.	Investigate the feasibility, efficacy, cosmetic results, and quality of life after single dose, ablative PBI. Tumor related genetic characteristics associated with radiotherapy responsiveness will be evaluated.	pCR	April 2018, ACTUAL
NCT02212860	Recruiting	Stereotactic Image-Guided Neoadjuvant Ablative Radiation Then Lumpectomy (SIGNAL)	Stereotactic Body Radiation delivered in prone position, using Volumetric-modulated arc therapy (VMAT), planned on co-registered PET/MRI and CT imaging. Dose: single dose of 21 Gy, then surgery.	Assess toxicity, cosmesis and efficacy of single fraction SBRT in early stage BC. A pathologic assessment of the impact of radiation at a microscopic level and on tumor markers will be performed.	Toxicity graded according to CTCAE 4.0	April 2018, ACTUAL

*List of abbreviations: BC* breast cancer, *CTV* clinical target volume, *DLT* dose limiting toxicity, *GTV* gross tumor volume, *MR* magnetic resonance, *MTD* maximum tolerated dose, *PBI* partial breast irradiation, *pCR* pathological complete response, *SBRT* stereotactic body radiotherapy, *RT* radiotherapy, *NS* not stated, *RTOG* Radiation Therapy Oncology Group, *EORTC* European Organisation for Research and Treatment of Cancer, *CTCAE* Common Terminology Criteria for Adverse Events

In the Canadian SIGNAL study (NCT02212860) [26] patients received a single 21 Gy fraction with external beam RT (i.e., VMAT) followed by definitive surgery 1 week later. No postoperative RT was planned. At 12 months after surgery toxicity, patient- and physician-rated cosmesis, and quality of life were not significantly different from baseline.

The attractiveness of the single dose has encouraged the start of other investigations, such as the study from the University of Florence (NCT03520894), delivering 21 Gy, with acute skin toxicity as primary endopoint and the one by Tiberi and coll. (NCT03917498) [27], where a dose of 20 Gy is given to the tumor with SBRT. The latter one is followed by BCS after 3 months and by a postoperative moderately hypofractionated WBRT in case of risk factors as G3 tumor, lymphovascular invasion, tumor size ≥3 cm, pT4 disease, triple-negative status, lobular histology, or extensive ductal carcinoma in situ (> 25% tumor mass). As a matter of fact, the optimal dose has not yet been established and dose-escalation studies continue to be designed. The single dose is currently being investigated in the phase I dose escalation study at the University of Texas Southwestern Medical center (NCT02685332), testing four dose escalation cohorts, 22.5 Gy, 25 Gy, 27.5 Gy and 30 Gy.

So far, there have been two phase II studies, both carried out in in the Netherlands, the Utrecht ABLATIVE study (NCT02316561) [25] and the Amsterdam PAPBI study (NCT01024582). Van Der Leij et al. published a preliminary report of the Amsterdam study [23] where the dose of 40 Gy in 10 daily fractions over 2 weeks was delivered to low risk BC patients with 3D CRT, IMRT or VMAT and no differences among techniques were reported.

The optimal timing to remove the irradiated tumor is another subject of debate. In the available literature (Tables 3 and 4), the RT-surgery interval ranges from 1 week (SIGNAL study) [26] to 6 months (ABLATIVE study) [25], which is still considered acceptable from patient’s perspective.

More insights are expected from the ongoing trials, especially from the Canadian SPORT trial (NCT01717261), where low risk BC patients receiving 20 Gy single fraction underwent either immediate surgery (24–72 h after RT, SPORT group) or delayed surgery (11–13 weeks after RT, SPORT -DS [delayed surgery] group). The authors observed a significant decrease in tumor cellularity in SPORT-DS cohort, while no change in cellularity occurred with immediate surgery, but a longer follow-up is needed to determine the prognostic significance of this finding.

Although the results remain limited in terms of number of cases and median follow-up time, the published data demonstrate the feasibility and the potential of preoperative APBI.

Our research project adds to the promising body of literature revolving around the use of stereotactic modality in single fraction in the preoperative setting for early-stage BC patients. The most ambitious goal is to identify radiological, biomolecular or genomic markers able to select good responders to RT for whom surgery could be omitted. Therefore, the preoperative phase is transitional in the development of the ablative treatment algorithm. Once knowledge and expertise have been consolidated, patients unfit for surgery due to advanced age or co-morbidities might be the ideal candidates, with the endpoint of progression free survival. In addition, APBI to the intact tumor might be offered in oligometastatic patients, alongside the treatment of the synchronous distant metastases, in order to treat all the sites of active disease.

## Data Availability

Data of this article will be not available until the final report of this study to avoid bias toward the analysis.

## Abbreviations

3D-CRT: Three-dimensional conformal RT
APBI: Accelerated partial breast irradiation
BC: Breast cancer
BCS: Breast conserving surgery
BRT: Brachytherapy
CT: Computed tomography
CTV: Clinical tumor volume
DLT: Dose limiting toxicity
GTV: Gross tumor volume
IMRT: Intensity modulated RT
MRI: Magnetic resonance imaging
MTD: Maximum tolerated dose
NGS: Next generation sequencing
PBT: Proton beam therapy
pCR: Pathological complete response
PTV: Planning tumor volume
RCB: Residual cancer burden
RT: Radiotherapy
SBRT: Stereotactic body RT
SRS: Stereotactic radiosurgery
VMAT: Volumetric-modulated arc-therapy
WBRT: Whole breast RT
